# Inductive Sensor Performance in Partial Discharges and Noise Separation by Means of Spectral Power Ratios

**DOI:** 10.3390/s140203408

**Published:** 2014-02-19

**Authors:** Jorge Alfredo Ardila-Rey, Mónica Victoria Rojas-Moreno, Juan Manuel Martínez-Tarifa, Guillermo Robles

**Affiliations:** Escuela Politécnica Superior, Departamento de Ingeniería Eléctrica, Universidad Carlos III de Madrid, Avenida de la Universidad 30, Leganés, Madrid 28911, Spain; E-Mails: jardila@ing.uc3m.es (J.A.A.-R.); jmmtarif@ing.uc3m.es (J.M.M.-T.); grobles@ing.uc3m.es (G.R.)

**Keywords:** partial discharges, noise separation, spectral power ratios, high frequency current transformers, inductive sensors

## Abstract

Partial discharge (PD) detection is a standardized technique to qualify electrical insulation in machines and power cables. Several techniques that analyze the waveform of the pulses have been proposed to discriminate noise from PD activity. Among them, spectral power ratio representation shows great flexibility in the separation of the sources of PD. Mapping spectral power ratios in two-dimensional plots leads to clusters of points which group pulses with similar characteristics. The position in the map depends on the nature of the partial discharge, the setup and the frequency response of the sensors. If these clusters are clearly separated, the subsequent task of identifying the source of the discharge is straightforward so the distance between clusters can be a figure of merit to suggest the best option for PD recognition. In this paper, two inductive sensors with different frequency responses to pulsed signals, a high frequency current transformer and an inductive loop sensor, are analyzed to test their performance in detecting and separating the sources of partial discharges.

## 1. Introduction

Electrical insulation failures are one of the most common causes of unexpected disconnection of electrical machines and power cables. The most expensive electrical assets in power generation, transmission and distribution are rated for high voltages, and are subjected to several electrical, thermal, environmental and mechanical stresses [1,2]. Among them, partial discharges are a common cause of failure, and, additionally, can reveal other degradation mechanisms [2].

Partial discharges are microscopic ionizations that occur in small insulation volumes due to non-homogeneous electrical fields and dielectric stresses. These partial dielectric breakdowns are limited within the insulation volume and involve low energy, so their immediate consequences are not catastrophic. However, physical and chemical attack of the surrounding solid or liquid insulation medium degrades the overall system until a final breakdown takes place [3,4]. Additionally, PD detection shows great capabilities to detect other degradation mechanisms inside electrical machines or power cables. For example, the analysis of phase resolved partial discharge (PRPD) patterns, which are pulses (in pC or mV) mapped together with the power-frequency applied voltage, can detect PD and noise and even identify the source of any PD that is active [5,6]. Moreover, the statistical treatment of these data can provide relevant information about the progress of degradation [6]. However, the signal-to-noise ratio (SNR) in the classical and band-limited (between 100 and 500 kHz) PD detection systems is usually very low because several noise sources (such as power electronic commutations) can be simultaneously active in industrial environments [7]. Despite the fact that there are other alternative techniques for PD detection [8,9], some of them are not compatible with the PRPD patterns.

In order to improve the PRPD recognition in noisy measurement setups, pulse waveform analyses have been proposed as a solution for PD filtering. In this sense, several pulse discrimination techniques, such as time-frequency maps [7], and wavelet filtering [10], have been implemented leading to good results in many experimental arrangements. Recently, the authors proposed the use of spectral power ratios (PR) maps as a more flexible technique for noise and partial discharge separation [11] obtaining good results as well. All these separation techniques require the use of broadband instrumentation systems (up to 100 MHz), which, most of the times, are based on inductive sensors in standard detection circuits, but with high bandwidths for pulse recognition. Among this kind of sensors, high frequency current transformers (HFCT) [7,11] and inductive loop sensors (ILS) [12,13], have been extensively used for PD detection.

In this paper, spectral power clustering techniques will be applied to several PD sources to analyze how these two typical inductive sensors are able to separate noise and PD pulses in the PR maps. Their performance has been quantified by the measurement of two different distances between clusters, obtaining significant results to select the best sensor for this classification technique.

## 2. Experimental Setup

**Figure 1.**
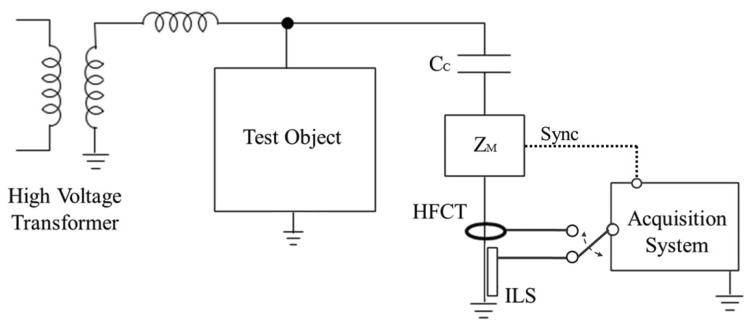
Experimental setup for PD measurements.

PD and noise pulses have been measured through the classical indirect detection circuit (see Figure 1) in a high-voltage laboratory [14,15]. A 750 VA testing transformer was used to create PD in several test objects. The coupling capacitor *Cc* (1 nF) is used as a low impedance path for high frequency currents from the PD pulses. The sensors were placed in the main ground conductor of this capacitive branch and sequentially connected to an acquisition system to record their different responses to the events. The measuring impedance *Zm* gives synchronization to the grid frequency so PD pulses can be plotted in conventional PRPD patterns.

The system used for the acquisition and processing of the pulses consists of an NI-PXIe-1082 chassis, an NI-PXI-5124 acquisition board with a sampling frequency of 200 MS/s and a resolution of 12-bit, and an NI-PXIe-8115 controller with a dual-core i5-2510E processor with 2 GB of RAM (all from National Instruments, Austin, TX, USA).

In the experiments, there is one test object for each type of discharge: a point near to a ground plane to create corona discharges, a ceramic bushing to create surface discharges and a stack of several insulating sheets of paper to obtain internal discharges. All of them displayed sustainable and controlled partial discharge activity some minutes after applying a high voltage.

**Figure 2.**
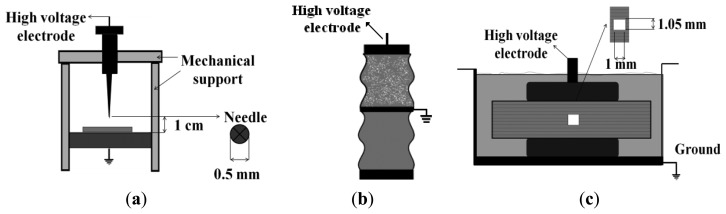
Devices under test: (**a**) Point-plane experimental specimen, (**b**) Contaminated ceramic bushing, (**c**) Pierced insulating sheets.

Corona discharges are the result of high divergent electric field spots, such as sharp metallic objects, where local ionizations in surrounding gas or air occur [14]. In this particular case, corona discharges were generated using a point-gas-plane setup, where the distance between the needle (5 mm in diameter) and the metallic ground plane is adjusted to 1 cm with a screw, as shown in Figure 2a.

Surface discharges occur along dielectric (air-metal-solid) interfaces where ionizations propagate orthogonally to the main applied field [14,16]. They can appear in bushings, outdoor insulators and in electrical machine end-windings. In this experiment, high voltage was applied to one of the connections of a ceramic bushing, see Figure 2b. Additionally, the insulator surface was contaminated with a saline solution, simulating a very common condition in coastal environments. A copper band was wrapped around and connected to ground in order to create shorter paths for surface discharges.

Internal PD are discharges that occur inside small voids in non-homogeneous solid dielectrics, usually containing gas, due to the differences in permittivity and dielectric strength [14]. The setup to generate internal discharges consists of eleven insulating sheets of NOMEX paper (polyimide 0.35 mm thick film; typically used in high voltage generators as slot insulation systems). Three of the central papers were pierced with a needle, creating an air hole (1 mm in diameter) with a circular shape. The overall layered dielectric was wrapped with a plastic envelope to create vacuum inside and the whole system was immersed in mineral oil to avoid surface discharges at low voltages, guaranteeing the generation of internal PD within the cylindrical void at moderate voltage levels (see Figure 2c).

For all test objects and with each sensor, noise, PD and the simultaneous acquisition of both phenomena are obtained following the procedure described in Table 1. The applied voltage is low when characterizing noise and high when acquiring PD. The differences in the applied voltage are determined by the partial discharge inception voltage (PDIV), which is different for each test object. The trigger level is low when capturing noise and high only when PD are acquired.

To obtain statistically significant results in each experiment, 1,000 pulses were acquired with one source (PD or noise) and 3,000 pulses were acquired when two simultaneous sources were active (PD and noise). This procedure guarantees the reliability of the observed phenomenon, since PD are ionization processes governed by the laws of statistical mechanics [3,4]. The procedure was repeated individually for each sensor, because their sensitivity is different (see Section 3) and the acquisition system only has two channels (one for the sensor and another for the synchronization signal) so the simultaneous measurement with the two sensors is impossible with this setup.

## 3. Inductive Sensors for PD Detection

**Figure 3.**
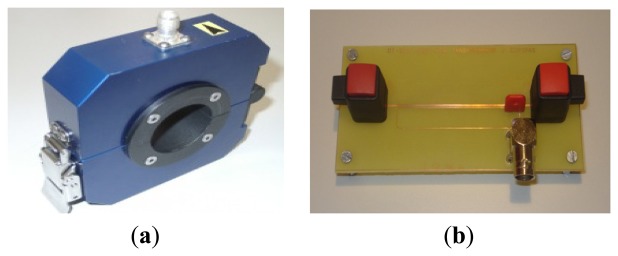
Two inductive sensors. (**a**) HFCT and (**b**) ILS.

Inductive sensors are common measuring devices for rapidly varying currents, such as partial discharges [17]. In this paper, a high-frequency current transformer and an inductive loop will be validated with the proposed spectral power ratios separation technique (explained in Section 4), see Figure 3.

The sensors' behavior is based on Faraday's Law. A PD produces a very short duration current pulse through the main ground conductor; this leads to the variation of the corresponding magnetic field which links the secondary of every sensor, coil or loop, and induces a voltage, *e*, proportional to the rate of change of the current, *i*. The proportional constant is *M*, the mutual inductance between the main conductor and the secondary, Equation (1). As the sensor is usually connected to a measuring system with a finite input impedance, the output voltage of each sensor is the induced voltage at low frequencies. However, at high frequencies, the effect of the device's self-inductance appears, and the sensor gives an output signal proportional to the magnitude of the current defining the sensor bandwidth:

(1) $$e=M\frac{di}{dt}$$

**Figure 4.**
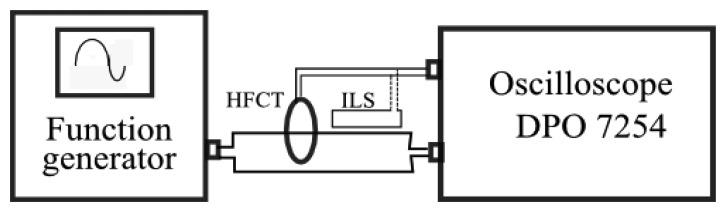
Experimental setups to obtain the frequency response of the sensors: HFCT and ILS.

The experimental frequency response of each sensor was obtained in the laboratory to characterize them completely. The setup consists of two circuits: the primary one is an arbitrary function generator Tektronix AFG 3252 connected to a channel of the oscilloscope with an input resistance of 50 Ω that serves as a load to the waveform generator. The secondary is the sensor itself: the coil in the HFCT or the loop in the ILS, that is connected to another channel, as can be seen in Figure 4. The generator creates a sinusoidal signal with amplitude of 2.5 V making sweeps in frequency from 1 MHz to 100 MHz. The empirical response of each sensor is calculated, dividing the output voltage of each sensor by the primary current that is obtained dividing the load voltage into 50 Ω.

### 3.1. HFCT

**Figure 5.**
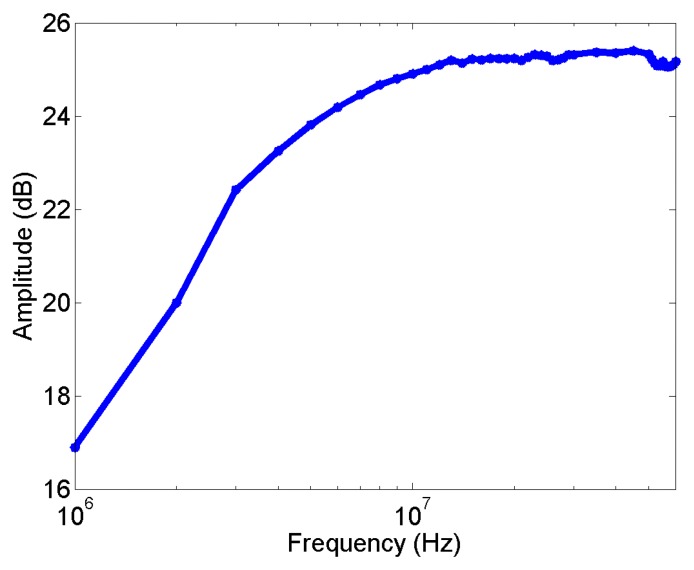
Experimental frequency response of the HFCT.

High-frequency current transformers are sensors with ferromagnetic cores so they have high sensitivity and high self-inductance and, in consequence, the output signals are proportional to the current at fairly low frequencies [18]. The experimental frequency response for the HFCT under test is shown in Figure 5. The results indicate that its sensitivity is around 25 dB and its bandwidth is approximately from 3 MHz (–3 dB low frequency) to 60 MHz.

### 3.2. Inductive Loop Sensor

**Figure 6.**
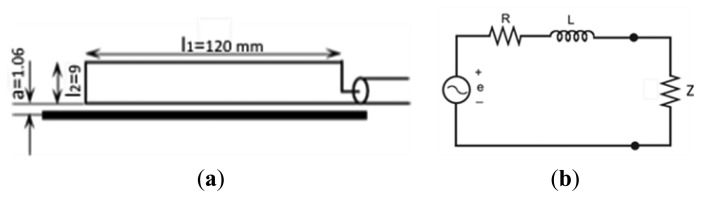
(**a**) ILS schematic and (**b**) its electric equivalent circuit.

This sensor consists of a conductor in the shape of a rectangular loop that is located close to a primary conductor where the PD pulse flows, Figure 6a. This can be modeled with the electric circuit of Figure 6b, where the induced voltage in the loop is represented by a source voltage *e*, which is in series with the resistance and the self-inductance of the loop, *R* and *L*, respectively. It is important to indicate that *Z*, usually 50 Ω, represents the input impedance of the measuring system, where the sensor is connected. More details relating the ILS theoretical model can be found in [13].

**Figure 7.**
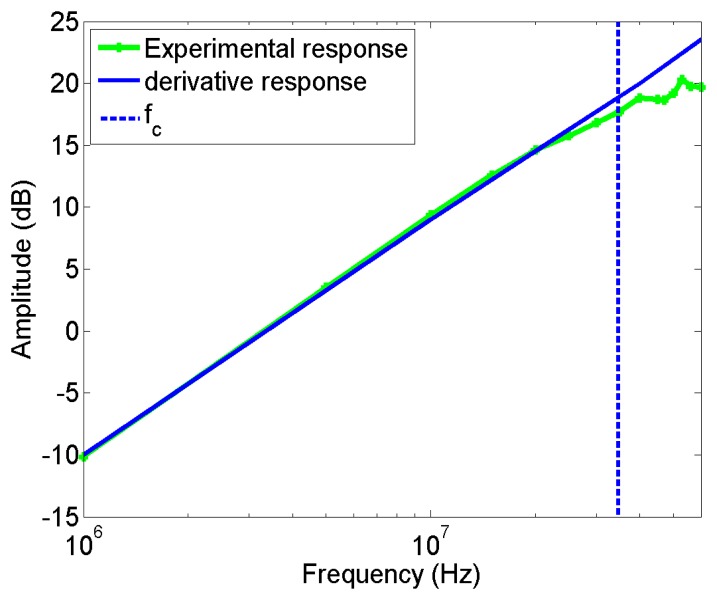
Experimental frequency and derivative response of the ILS.

The prototype used in this paper has the geometry and electrical parameters indicated in Table 2. They give a frequency response according to Equation (2) and to the equivalent circuit, which has a corner frequency, *f_c_*, indicated and calculated in Equation (3). This frequency limits the derivative response of sensor. This result is corroborated with the experimental response, see Figure 7, where the data coincide with the straight line, with a slope of 20 dB/decade, until f_c_,(vertical dotted line), where the sensitivity reaches 17.5 dB approximately, lower than that of the HFCT. For higher frequencies the amplitude of the response is further increased, but with a lower slope.

(2) $$\frac{V_{\text{out}}\left(s\right)}{I\left(s\right)}=\frac{\text{ZMs}}{Ls+R+Z}\approx \frac{\text{ZMs}}{Ls+Z}$$

(3) $$f_{c}=\frac{Z}{2\pi L}=\frac{50}{2\pi \cdot 229.4\cdot 10^{-9}}=34.69\text{MHz}$$

## 4. The Power Ratio Map

Past works have showed that, for the same detection circuit, the spectral energy at certain frequency bands is not the same for pulses of different PD types (surface, internal and corona) and noise. This has provided helpful information for the development of new techniques for separation and identification of different PD sources and noise [11].

**Figure 8.**
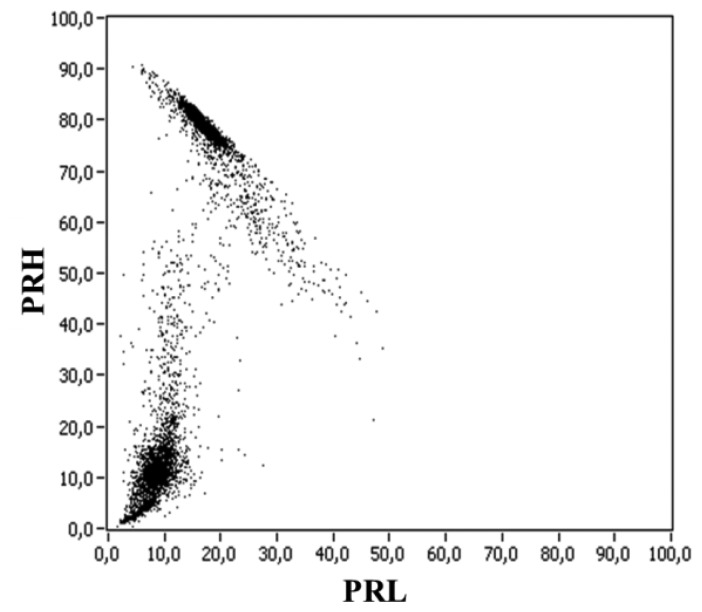
Power ratio representation [11].

Reference [11] presents the spectral power clustering technique, which is based on a two-dimensional map for power ratios (PR map), representing the relative spectral power content in two frequency intervals: PRL (power ratio for low frequencies) and PRH (power ratio for high frequencies), see Figure 8. To obtain these parameters, the Fast Fourier Transform (FFT) of each pulse is calculated and the spectrum is divided into two frequency intervals that are explicitly represented in a plane. The cumulative spectral power calculated for the two frequency intervals is normalized to the total spectral power. The obtained quantities are defined as power ratios (%), one for the higher frequency interval, PRH, and another for the lower frequency interval, PRL, as shown in Equations (4) and (5).

(4) $$\text{PRL}=\frac{{\text{∑}}_{f_{1L}}^{{\text{f}}_{2\text{L}}}\;{\left|s(f)\right|}^{2}}{{\text{∑}}_{0}^{f_{T}}\;{\left|s(f)\right|}^{2}}\cdot 100$$

(5) $$\text{PRH}=\frac{{\text{∑}}_{f_{1H}}^{f_{2H}}\;{\left|s(f)\right|}^{2}}{{\text{∑}}_{0}^{f_{T}}\;{\left|s(f)\right|}^{2}}\cdot 100$$

where:

- *s(f)* is the magnitude of the FFT of the pulse signal, *s(t).*
- [*f_1L_, f_2L_*] corresponds to the lower frequency interval represented.
- [*f_IH_, f_2H_*] corresponds to the higher frequency interval represented.
- *f_T_*, is the maximum frequency under analysis.

There are two important aspects that are necessary to take into account: first, the conditions 0 ≤ f_1L_ < f_2L_, f_1H_ < f_2H_ ≤ f_T_ and f_1L_ < f_2H_, are mandatory; second, the frequency intervals can be changed depending on the spectral power distribution of the detected signals, and they can be complementary (if f_1H_ ≥ f_2L_) or overlapping (if f_1H_ < f_2L_), which gives versatility for this technique.

Paper [11] shows that, using a HFCT, partial discharges and noise have different values of PRH and PRL, and are grouped in specific regions of the map, which may help in the identification of pulse sources and, therefore, in the diagnosis of the insulation system. The system operator could select a cluster of points from the maps and represent the associated PRPD, which would give the final source identification, once the frequency intervals that define the low-frequency and high-frequency regions are chosen. It is clear that the selection of one cluster is simpler if it is well separated from the others in the map. This can be achieved by selecting the most appropriate hardware (sensor). The positions of the clusters in the map are not definitive and, as mentioned in [11], different experimental configurations or industrial environments might lead to changes in the location of the clusters.

## 5. Processing the PD Data

**Figure 9.**
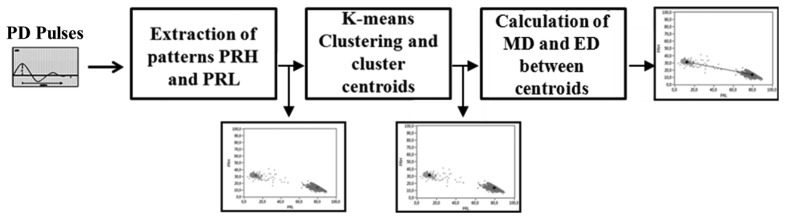
Schematic diagram of the procedure.

As described in Section 3, each sensor has a different behavior in the frequency domain according to its design, therefore the location and shape adopted by the clusters in the PR maps will depend not only on the selection of the frequency intervals for PRL and PRH, the equivalent capacitance of the test object, and, of course, on the pulse nature, but also on the type of sensor used. The procedure used to quantify the separation capability of each sensor is summarized in Figure 9. Once the clusters associated with PD and noise are plotted in the PR maps, a K-means algorithm is used to identify these clusters with their centroids automatically. Subsequently, the distance between the centroids in each map is quantified in two ways:

- *Euclidean distance (ED)*: Calculates the distance between the centroids without regard the location of the points within each cluster.
- *Mahalanobis distance (MD)*: Calculates the distance between the centroids taking into account the homogeneity of the clusters *i.e.*, the dispersion of the points within a cluster from its centroid.

### 5.1. K-means Clustering

K-means is a clustering method used to find and identify clusters and centroids of clusters in a set of unlabeled data. It is considered as an efficient method for these applications since it can analyze numerous variables and samples avoiding the use of too much memory space. In addition, its processing speed is very fast [19,20].

For example, a set of n objects *X_i_*, *i* = 1, 2, …, *n*, is partitioned into *k* groups whose centers are *C_j_*, *j* = 1, 2, …, *k*. The objective function *J*, based on the distance between an object *X_i_* in group *j* and the corresponding cluster centroid *C_j_*, can be defined by:

(6) $$J=\overset{k}{\underset{j=1}{\text{∑}}}\overset{n}{\underset{i=1}{\text{∑}}}{\left\|X_{i}-C_{j}\right\|}^{2}$$

The K-means algorithm randomly selects k of the given objects to represent the cluster centroid. Based on the selected objects, all remaining objects are assigned one by one to their closer centroid. The distance between the object and every centroid is computed, and then, the object is assigned to the cluster which yields the minimum distance. The value of the selected centroid is recalculated by taking the mean of all the points belonging to the same cluster. The operation is iterated for all the objects. The same procedure is repeated until the objective function converges (when there are no objects that are exchanged between groups). If k cannot be known a priori, various values of k can be evaluated until the most suitable one is found. The effectiveness of this method, as well as of others, relies heavily on the objective function used in measuring the distance between objects [21,22].

Concretely, the steps of the k-means algorithm can be summarized in:

1. Initialize the clusters centroids.
2. Group each data point into the nearest cluster based on distance calculation.
3. Recalculate each center using the mean of all the points in the same cluster.
4. Move some data points from one cluster to another to make J smaller, and return to step 2 until the convergence condition is met.

### 5.2. Euclidean Distance

The most widely used distance is the ED. It is simple computationally, but this parameter does not consider the structure of the data [23,24]. The Euclidean distance between any two clusters of data, A and B, is the distance between their centroids and is given by:

(7) $$ED^{2}={\left({\text{C}}_{A}-{\text{C}}_{B}\right)}^{\text{T}}\left({\text{C}}_{A}-{\text{C}}_{B}\right)$$

where, *C_A_* and *C_B_* are centroids of the clusters *A* and *B*. In this particular case, *(C_A_* − *C_B_)* is a vector, since the representation of the points is done in two dimensions (PR maps).

### 5.3. Mahalanobis Distance

The ED is often invalid and generally undesirable in many practical applications since it ignores helpful parameters about the data, such as homogeneity or dispersion. Thus, the MD is better in this aspect because it includes the variances of the data as weights to modify the distance [23,25].

The MD between two clusters of data, A and B, is given by:

(8) $$MD^{2}={\left({\text{C}}_{A}-{\text{C}}_{B}\right)}^{\text{T}}\left({V_{A}}^{-1}-{V_{B}}^{-1}\right)\left({\text{C}}_{A}-{\text{C}}_{B}\right)$$

where, *V_A_* and *V_B_* represent the covariance matrices computed from of *A* and *B*, Equations (9) and (10).

(9) $$V_{A}=\frac{1}{{\text{N}}_{\text{A}}}*\overset{{\text{N}}_{\text{A}}}{\underset{j=1}{\text{∑}}}{\left(A_{j}-{\text{C}}_{A}\right)}^{\text{T}}\left(A_{j}-{\text{C}}_{A}\right)$$

(10) $$V_{B}=\frac{1}{{\text{N}}_{\text{B}}}*\overset{{\text{N}}_{\text{B}}}{\underset{j=1}{\text{∑}}}{\left(B_{j}-C_{B}\right)}^{T}(B_{j}-C_{B})$$

where, *N_A_* and *N_B_* are number of data within each cluster.

## 6. Experimental Results

This section presents the results quantifying the separation capability between clusters associated with PD sources and noise. These are obtained by applying the spectral power clustering technique to the signals measured in different test objects with the HFCT and ILS sensors. The PR maps and the normalized average spectral power (it is has been normalized in such a way that its spectral peaks are always lower than 1) of the pulses captured by two sensors are described in all the test objects. Due to the fact that the HFCT response has higher sensitivity, 25 dB and it is proportional to the current pulse in the bandwidth of [3–60] MHz (see Figure 5), the authors have chosen the frequency intervals based on the observation of the spectra obtained with this sensor in all the experiments (taking into account the bands with higher spectral power for its response). For all measurements, the frequencies for PRH and PRL calculation were set to: *f_1L_* = 10 MHz, *f_2L_* = *f_1H_* = 30 MHz, *f_2H_* = 50 MHz, *f_T_* = 60 MHz (see dotted lines in the figures below). Thus, complementary frequency intervals were chosen for PRH and PRL and two intervals are not included in the PR maps: a low frequency interval, [0, 10] MHz, and a high frequency interval, [50, 60] MHz. The selection of the same frequency ranges for PRH and PRL calculation (20 MHz) ensures that both parameters have the same cumulative capability for pulse representation in the PR map.

### 6.1. Corona Discharges

**Figure 10.**
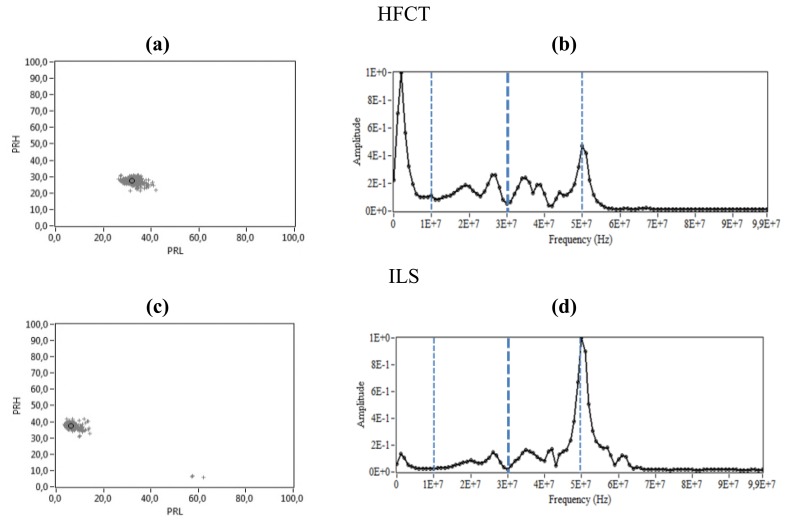
Corona PD. (**a**) PR map of the signals measured with the HFCT, (**b**) Normalized average spectral power of the signals measured with the HFCT, (**c**) PR map of the signals measured with the ILS, (**d**) Normalized average spectral power of the signals measured with the ILS.

For this experiment, the voltage applied to the point–plane setup was 5 kV. The clusters and the normalized average spectral power captured by each sensor in this experiment are shown in Figure 10. There are two maximum peaks of power in the signal obtained with the HFCT, in 2 MHz and 50 MHz, which are also captured by the ILS in each of the measurements (see Figure 10b,d). However, while the power captured in 2 MHz by the HFCT has high amplitude, the ILS barely detects it, due to the fact that this sensor has a low gain in the units of MHz, as can be observed in its experimental frequency response presented in Figure 7.

The second peak is clearly detected by both sensors, being the maximum peak in the ILS spectra because it gives output signals with high amplitudes around the tens of MHz. In the PR maps (Figure 10a,c), the position of the clusters is coherent with the values observed in the normalized average spectral power for each sensor. The spectral power content in the interval [10, 30] MHz, (used for PRL calculation), is higher for the HFCT and lower for the ILS, which is confirmed by the position of the clusters for each sensor in the PRL axis. As expected for the interval [30, 50] MHz, the ILS has higher values for PRH. In Figure 10c, a small group of points appears clearly at the bottom of the PR map, which are associated with some type of noise source of large amplitude present during this acquisition, which is confirmed in the next section.

### 6.2. Surface Discharges

**Figure 11.**
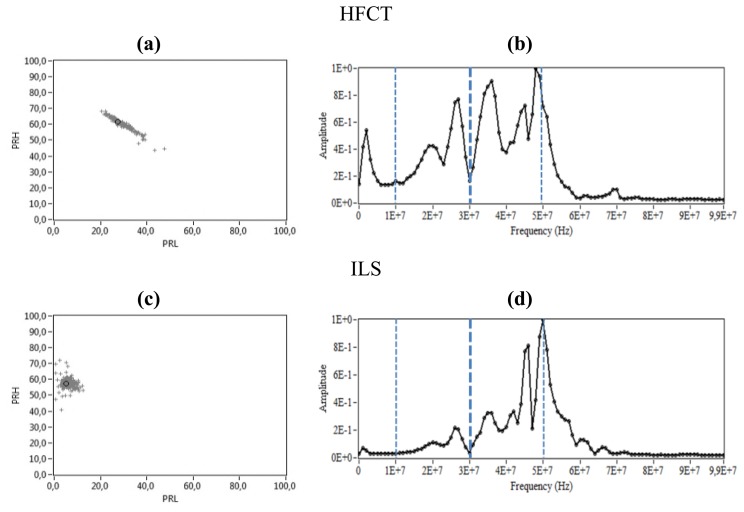
Surface PD. (**a**) PR map of the signals measured with the HFCT, (**b**) Normalized average spectral power of the signals measured with the HFCT, (**c**) PR map of the signals measured with the ILS, (**d**) Normalized average spectral power of the signals measured with the ILS.

Figure 11 represents the PR maps and the normalized average spectral power of the signals measured by applying 8.3 kV to the ceramic bushing.

In this case, the spectral power components detected by the HFCT and ILS in the interval [10, 60] MHz is higher than those obtained in the previous experiment, which is characteristic of this type of discharges, Figure 11b,d. Both sensors detect a maximum peak of power around 50 MHz similar to that obtained with corona PD, but this time more meaningful for the HFCT (compared to its low-frequency response for corona). Additionally, there is a high power concentration between 25–40 MHz, sequentially growing for ILS with the increasing in frequency, as expected according to its frequency response. The position of the clusters in the PR maps for this type of discharges, Figure 11a,c, again coincides with the spectral power content in the high and low frequency intervals of Figure 11b,d, where, PRL is higher for the HFCT while PRH is similar for both.

### 6.3. Internal Discharges

**Figure 12.**
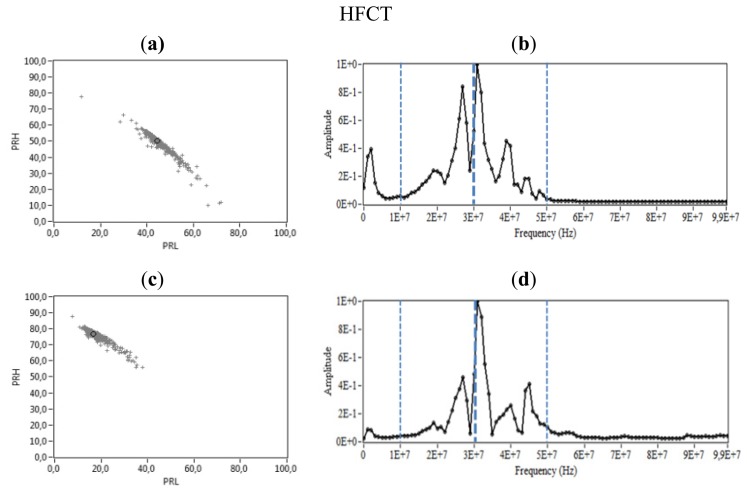
Internal PD. (**a**) PR map of the signals measured with the HFCT, (**b**) Normalized average spectral power of the signals measured with the HFCT, (**c**) PR map of the signals measured with the ILS, (**d**) Normalized average spectral power from the signals measured with the ILS.

Figure 12 shows the PR maps and the normalized average spectral power of the signals measured by applying 9 kV to the test specimen described in Section 2. This type of discharges has a high spectral power in the interval [10, 60] MHz and lower in the interval [0, 10] MHz, so this PD source shows some similarities with surfaces discharges and few with corona PD. However, internal discharges obtained with this test object differ from other types of PD (corona and surface discharges) because they do not have a high magnitude component at 50 MHz, Figure 12b,d. For the HFCT and ILS, the maximum peaks of power are located between 20–40 MHz, finding the largest spectral power component close to 30 MHz for both sensors. The clusters associated with this type of discharges present high dispersion of spectral power in PRL and PRH. The cluster obtained with the HFCT has values of PRL higher than that of the ILS. The highest values of PRH were obtained with the ILS (see Figure 12a,c), which is in agreement to spectral power shown in Figure 12b,d and with its experimental frequency response, see Figure 7.

### 6.4. Separation Capability of PD Sources and Noise

**Figure 13.**
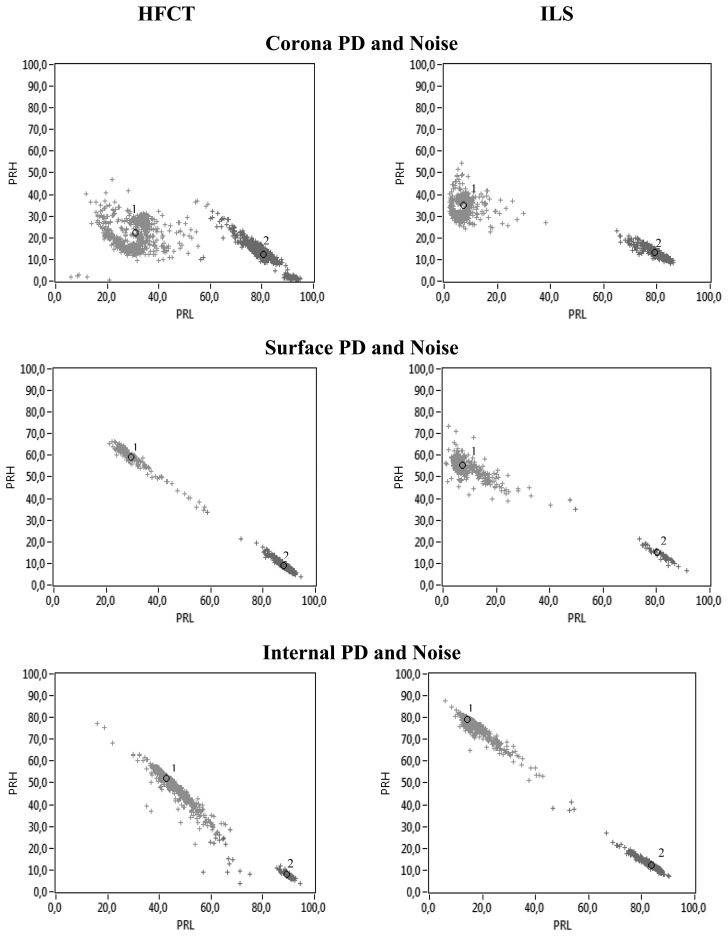
PR maps for PD (cluster 1) and noise (cluster 2) obtained with both sensors.

The following step was the processing of data from the experiments where partial discharges and noise were simultaneously active. Figure 13 shows the clusters obtained with their respective centroids for the HFCT and ILS sensors. Both sensors show two clusters clearly different for all the experiments, so the identification of PD is possible when the associated PRPD for each PD cluster is represented.

**Figure 14.**
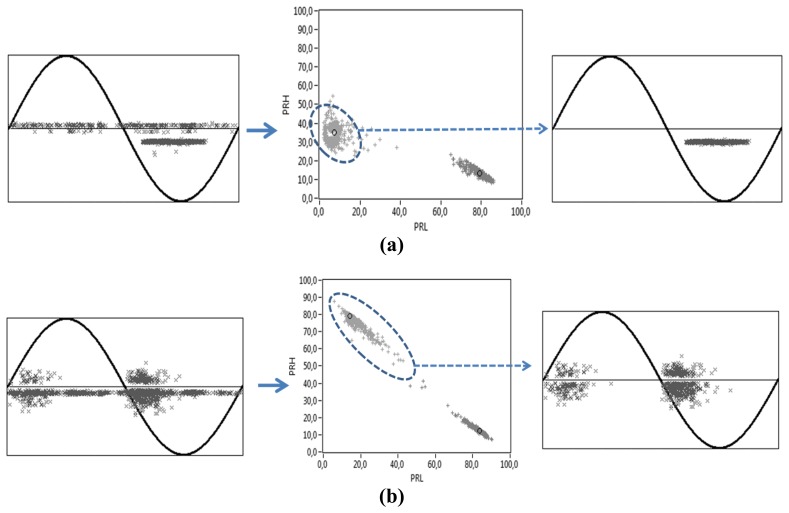
Complete PRPD pattern (Left), PR map (Center) and filtered PRPD (Right) obtained with the ILS sensor for (**a**) corona discharges and (**b**) internal discharges.

For example, in the critical case of the PR maps obtained with the ILS, which has lower SNR, corona and internal discharges can be separated from the noise signals if one of the clusters is selected and represented in the PRPD graph. For this case, Figure 14a shows the associated PRPD pattern for the selected cluster, obtaining the typical PRPD for corona discharges, where highly stable PD magnitudes are observed for the negative maxima of the applied voltage [5]. Likewise, Figure 14b shows the PRPD pattern from internal discharges, where high-magnitude discharges occur in the phase positions where the voltage slope is maximum [5]. In the original PRPD patterns (left part of Figure 14), the uncorrelated pulses in phase represent the captured electrical noise during the acquisition and they are clearly characterized as a cluster in the lower right part of the PR maps (high spectral power in the represented interval [10, 30] MHz and low spectral power in the interval [30, 50] MHz). It is clear that the complete PRPD pattern would have led to important errors assessing the statistical magnitude and repetition rate of the PD activity. Therefore, a good separation between the clusters ensures an appropriate identification of the sources present during the measurement. This was the procedure to identify the clusters of Figure 13 as PD (cluster 1) or noise (cluster 2) for all the test objects.

Table 3 summarizes the results obtained in terms of separation between PD and noise clusters shown in Figure 13 with both sensors. The comparison is made taking the values obtained with the HFCT as reference. As far as the Euclidean distance is concerned, the ILS shows the best separation between clusters for all cases (corona, surface and internal PD) giving a separation 46.33% higher than that obtained with the HFCT for the case of corona PD, 8.66% higher for surface PD and 63.57% for internal PD.

Bear in mind that the ED does not take into account the cluster dispersion, which could lead, in the worst case, to clouds nearly overlapping, as described in Section 5. This is why the Mahalanobis distance was also considered, since it provides a value accounting for the statistical quality of the cluster (lower dispersion of the PR clusters leads to better separation capability). The MD results are not so categorical for the three experiments, because the ILS separates better in the corona (159.56% higher) and internal PD (135.10% higher) experimental setups, but in the surface discharges case, the distance between clusters is 4.74% lower, and the HFCT becomes the best option, though the difference is small. In general, both sensors are able to separate clusters associated with PD and noise clearly in the PR maps. However, it seems that the derivative characteristic of the ILS, gives better results for PD and noise separation for this simple inductive sensor.

With the aim of checking if the better performance of ILS *versus* HFCT in PD and noise source separation prevails, two new frequency sets were tested. For the first set, the frequencies were set to: *f_1L_* = 0 MHz, *f_2L_* = *f_1H_* = 20 MHz, *f_2H_* = 40 MHz, *f_T_* = 60 MHz, for the second set, the frequencies for PRH and PRL calculation were set to: *f_1L_* = 20 MHz, *f_2L_* = *f_1H_* = 40 MHz, *f_2H_* = 60 MHz, *f_T_* = 60 MHz. This selection still uses frequency ranges of 20 MHz for PRH and PRL calculations, but modifying their “central frequencies” to different positions within the observed bandwidth of interest (0–60 MHz).

The ED and MD obtained with the first interval for both sensors are shown in Table 4. The ED values were higher for ILS in the surface PD case (92.38%) and internal PD (15.65%), but lower for corona PD (14.48%). Regarding the MD, the results indicate that were considerably higher for the ILS in the three experiments: 121.75% for corona PD, 145.25% for surface PD and 104.21% for internal PD, which means that for this interval the clusters obtained with ILS have a far lower dispersion than those obtained with the HFCT. The worse behavior of the ILS for corona and noise separation using ED is related to the lost of spectral power for calculations for the [40, 60] MHz interval, where a high relative peak was present for corona PD (see Figure 10).

Table 5 indicates the ED and MD values obtained for the second interval. In this case, the ED and MD were much higher for the ILS in every experiment. These results confirm the low dispersion of the clusters that are obtained with the ILS, and its high separation capacity for different intervals. In this case, the PR calculations are focused in frequency intervals where the ILS gives more spectral power (see Figures 10,11–12), so its corresponding points will be placed at positions further away from the PR origin.

## 7. Summary

**Figure 15.**
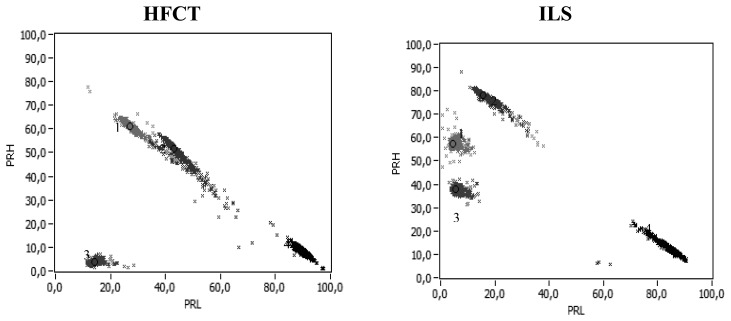
Summary of the relative positions of the HFCT and ILS sensors in the PR maps to *f_1L_* = 10 MHz, *f_2L_* = *f_1H_* = 30 MHz, *f_2H_* = 50 MHz, *f_T_* = 60 MHz. Surface PD (cluster 1), Internal PD (cluster 2), Corona PD (cluster 3) and Noise (cluster 4).

Figure 15 is the summary of the results obtained in the three experiments that are detailed along this paper. The clusters associated with PD or noise are simultaneously shown for the ILS and HFCT sensors.

In each of the maps, a single cluster of noise is represented, since it was observed that the parameters PRL and PRH of the clusters associated with noise exhibit similar positions in the PR maps regardless of the test object. The clusters associated to the three PD sources for the two sensors are located in different regions of the classification map, which is a great advantage in case of several PD sources and noise acting simultaneously. In the ILS case, the relative separation between PD and noise clusters and between the clusters associated with different partial discharges (surface, internal and corona) is most notorious. Moreover, in the results of the HFCT, the relative separation between the clusters 1 and 2 is not obvious, since both clusters almost coincide in the same region of the map. (Please note this paragraph has been divided into 2: Answer: we agree with this change)

## 8. Conclusions

This paper shows that the power ratios map is a successful separation technique for PD identification when the HFCT or ILS sensors are used for detection. The inductive loop sensor leads to the best cluster separation for noise and partial discharges in seven out of nine of the experiments with different test objects and frequency intervals thanks to its derivative behavior up to tens of MHz. When it works worse, the difference in performance compared to the current transformer is negligible. This is an interesting characteristic when PD detection is made in industrial environments, where noise filtering is a challenge for insulation diagnosis: a better cluster separation allows detecting PRPD patterns with easier interpretation. High frequency current transformers work properly with this spectral power analysis strategy, but with lower features than ILS in most of the cases; however, its higher sensitivity makes them useful in sites where SNR is poor.

## Figures and Tables

**Table 1.** Trigger and voltage levels to characterize noise, PD and PD + noise.

**Characterization**	**Applied Voltage**	**Trigger Level**
Noise	Low	Low
PD	High	High
PD and noise	High	Low

**Table 2.** Parameters of the used ILS.

**l_1_ [mm]**	**l_2_ [mm]**	**a [mm]**	**M [nH]**	**R [Ω] to 10 MHz**	**L [nH]**	**Z [Ω]**
120	9	1.06	54.9	0.22	229.4	50

**Table 3.** MD and ED between clusters obtained with the two sensors for the intervals PRL [10, 30] MHz and PRH [30, 50] MHz.

**Corona PD and Noise**				
Sensors	ED		MD	
HFCT	52.25	%	7.32	%
ILS	76.46	46.33	19.0	159.56
Surface PD and Noise				
HFCT	77.02	%	16.44	%
ILS	83.69	8.66	15.66	−4.74
Internal PD and Noise				
HFCT	57.22	%	7.52	%
ILS	93.60	63.57	17.68	135.10

**Table 4.** MD and ED between clusters obtained with the two sensors for the intervals. PRL [0, 20] MHz and PRH [20, 40] MHz.

**Corona PD and Noise**				
**Sensors**	**ED**		**MD**	
**HFCT**	61.80	**%**	6,85	**%**
**ILS**	52.85	−14.48	15.19	121.75
**Surface PD and Noise**				
**HFCT**	26.79	**%**	5.90	**%**
**ILS**	51.54	92.38	14.47	145.25
**Internal PD and Noise**				
**HFCT**	21.97	**%**	4.74	**%**
**ILS**	25.41	15.65	9.68	104.21

**Table 5.** MD and ED between clusters obtained with the two sensors for the intervals. PRL [20, 40] MHz and PRH [40, 60] MHz.

**Corona PD and Noise**				
Sensors	ED		MD	
HFCT	51.97	%	6.90	%
ILS	85.09	63.72	15.19	120.14
Surface PD and Noise				
HFCT	43.46	%	15.43	%
ILS	82.15	88.67	17.47	13.22
Internal PD and Noise				
HFCT	16.42	%	4.53	%
ILS	37.86	130.57	9.16	102.20
